# Mn-ZIF nanozymes kill tumors by generating hydroxyl radical as well as reversing the tumor microenvironment

**DOI:** 10.3389/fphar.2024.1441818

**Published:** 2024-08-13

**Authors:** Jiyu Han, Hairong Ma, Songtao Ai, Daqian Wan

**Affiliations:** ^1^ Department of Orthopedics, Tongji Hospital, School of Medicine, Tongji University, Shanghai, China; ^2^ Key Laboratory of Spine and Spinal Cord Injury Repair and Regeneration, Ministry of Education, Shanghai, China; ^3^ Department of Radiology, Shanghai Ninth People’s Hospital, Shanghai Jiao Tong University School of Medicine, Shanghai, China

**Keywords:** Mn-ZIF nanozymes, hydroxyl radical, fenton reaction, macrophage polarization, combined tumor therapy, osteosarcoma

## Abstract

Tumor tissues are well known for their unique high hydrogen peroxide (H_2_O_2_) microenvironment. How to exploit this tumor microenvironment for tumor cell killing is a question. In this study, a Mn-doped metal-organic framework (Mn-ZIF) was constructed. It possesses good peroxidase (POD) activity, which can oxidize tumor-localized H_2_O_2_ into hydroxyl radicals (·OH), that possesses the ability to directly kill tumor cells. More surprisingly, *in vivo* experiments the researchers not only observed the tumor-killing effect of Mn-ZIF, but also found it changes in macrophage phenotype in the tumor region. There was an increase in macrophage polarization towards the M1 subtype. This suggests that the tumor-killing effect of Mn-ZIF not only comes from its POD activity, but also regulates the immune microenvironment in the tumor region. In conclusion, the preparation of Mn-ZIF provides a new way for comprehensive tumor therapy.

## Introduction

Tumor regions are known for their special microenvironment. For example, localized low pH, high glutathione (GSH), and high H_2_O_2_ (Feng et al., 2017; Zhang et al., 2023b). This is associated with the rapid proliferation of tumor cells and metabolic characteristics such as anaerobic respiration. This is known as the “Warburg effect.” The poor tumor microenvironment further inhibits the growth of normal cells and the body’s immune cells to play their proper functions, assisting the vicious cycle of tumor growth (Yang et al., 2021). Among them, H_2_O_2_ is a kind of reactive oxygen species (ROS), which can be used as a signaling molecule to participate in the processes of cell proliferation, angiogenesis and immune escape (Martínez-Reyes and Chandel, 2021). So how to achieve its consumption while killing tumor cells is a challenge.

The immune microenvironment in the tumor region was also affected. Most notably, macrophage polarization. Macrophages are known to polarize more toward the M2 subtype in the tumor region, creating a microenvironment that promotes tumor growth. At the same time, the proportion of macrophages of the M1 subtype that inhibit tumor growth is reduced (van Elsas et al., 2024; Xiao et al., 2024). As the most infiltrated immune cells in the osteosarcoma region, this effect of macrophage polarization is particularly prominent (Wang et al., 2022). If this poor direction of differentiation can be reversed, tumor development and metastasis can be effectively inhibited.

Metal-organic frameworks provide a new approach for the regulation of the tumor microenvironment. Zeolite imidazolium salt framework (ZIF) is a nanostructure that possesses the advantages of hollow mesopores and large specific surface area (Liu et al., 2024; Xu et al., 2024). Previous researchers have exploited these advantages for chemotherapeutic drug piggybacking and constructed a variety of nano-delivery systems in the tumor region (Wang et al., 2024a). In addition, the numerous active sites on its surface provide a great convenience for its modification. A modified ZIF structure was introduced to provide more bioactivity itself (Zhang et al., 2023a; Wang et al., 2024b).

The development of Mn-containing nanoparticles as antitumor drug carriers has gradually stepped into the limelight in recent years. They can promote the inflammatory response of macrophages by generating reactive oxygen species and activating the NF-κB signaling pathway, thus enhancing the killing effect on osteosarcoma cells (Liang et al., 2023; Zhu et al., 2023). Mn-containing nanoparticles have important research value and application potential in regulating macrophage polarization, which can provide new strategies and methods for the treatment of diseases.

**SCHEME 1:**
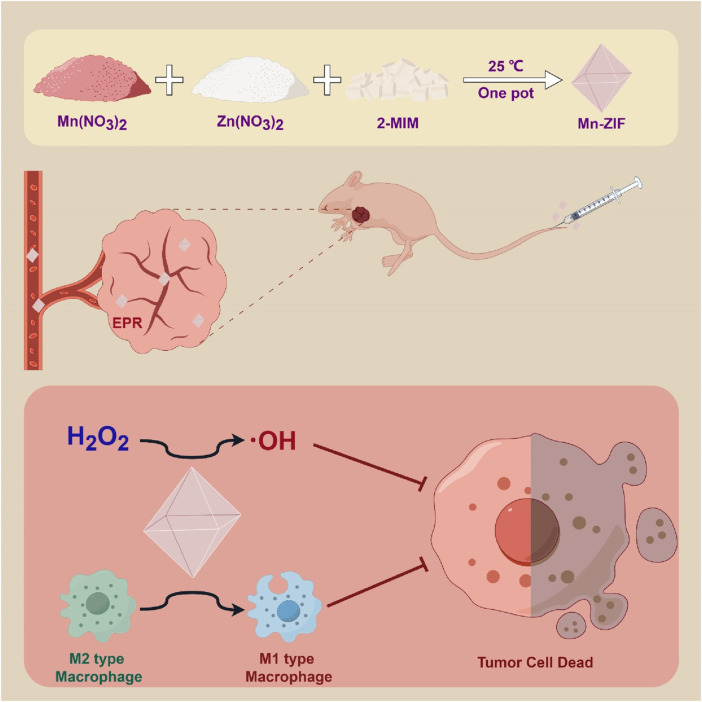
Utilization of Mn-ZIF nanozymes for the purpose of anti-cancer therapy.

In this paper, we constructed Mn-ZIF nanozymes. Its potent POD activity was demonstrated *in vitro*, as well as its ability to generate tumor-killing ·OH via the Fenton reaction. Its immune microenvironment reprogramming ability and tumor-killing capacity were subsequently validated in a nude mouse subcutaneous osteosarcoma model. This strategy provides a simple and efficient idea for the comprehensive treatment of tumors (Scheme 1).

## Materials and methods

### Synthesis and characterization of Mn-ZIF

Zinc-based zeolitic imidazolate framework (ZIF) was prepared by dissolving Zn(NO_3_)_2_·6H_2_O in N, N-dimethylformamide (DMF) and adding to DMF solution. The resulting precipitate was collected by centrifugation. Manganese-doped ZIF (Mn-ZIF) was prepared by dissolving Mn(NO_3_)_2_·6H_2_O and Zn(NO_3_)_2_·6H_2_O in DMF and stirring the mixture. Subsequently, a dimethylformamide solution containing dimethylimidazole was added. The resulting mixture was stirred, followed by collection of the precipitate, washing thrice and vacuum drying.

### Mouse grouping, treatment, and tumor volume measurement experiment

A total of 12 mice with a tumor volume of 80 mm^3^ tumor area were randomly assigned to Control, ZIF, and Mn-ZIF groups. Via tail vein injection every 4 days for 4 doses. The treatment doses administered in this study as follow: Con-200 μL saline. ZIF-200 mg/kg of ZIF. Mn-ZIF-200 mg/kg of Mn-ZIF. The experiment concluded on 16th, during tumor was assessed using the formula V = 4π/3ab2, where a and b (mm) denote the long and short tumor diameters, respectively. Upon completion, mice were humanely euthanized. Subsequently, the major organ were harvested. The tumor areas were photographed and weighed. The Medical Ethics Committee of Tongji Hospital Affiliated to Tongji University, approved all experimental procedures.

### Flow cytometry

A sufficient quantity of tumor tissue was obtained and adequately processed, followed by digestion with a solution containing type IV collagenase to achieve a single cell suspension. The cells were then filtered through a cell strainer into a centrifuge tube, where tumor cells were enriched for lymphocytes. Subsequently, the cells were quantified and a range of 5 × 10^5^–1 × 10^6^ cells were isolated into a flow-through tube and stored at 4°C. After centrifugation at 1,650 r/min for 5 min, the supernatant was discarded. Following this, 200 μl of FACS solution was added, along with the appropriate volume of antibodies targeting surface antigen molecules such as CD45, excluding CD206. Incubate samples at 4°C in the absence of light for 30–60 min. Resuspend cells by adding 2–5 mL of ice-cold FACS solution, followed by centrifugation at 1,650 revolutions per minute for 5 min to remove the supernatant and ensure no residue remains (this step may be repeated twice). Subsequently, add 200 μL of Foxp3 Fixation/Osmotic Stabilization Working Solution (3:1) to each tube, pulse vortex, and incubate at 4°C for a minimum of 60 min away from light. Without washing, add 2 mL of 1x Permeabilization solution to each tube. Centrifuge the samples at 1,650 revolutions per minute for 5 min at 4°C and discard the supernatant. Resuspend the precipitate in 100 μL of 1X Permeabilization solution and add the recommended amount of fluorescent dye-coupled antibody CD206 for the detection of intracellular cellular antigens. Incubate the mixture for 30–60 min at 4°C in the absence of light. Subsequently, add 2 mL of 1X Permeabilization solution to each tube. The samples were centrifuged at 1,650 revolutions per minute for 5 min at 4°C, followed by discarding the supernatant. The stained cells were then resuspended in 300 μL of FACS buffer and analyzed using a flow cytometer.

The flow cytometry cell-circle gating pattern for M1 and M2 macrophages was characterized by the following markers: M1 macrophages were labeled as CD45^+^, F4_80+, CD11b+, MHC II+, CD206-, while M2 macrophages were labeled as CD45^+^, F4_80+, CD11b+, MHC II-, CD206+.

## Results and discussion

### Preparation and characterization of ZIF and Mn-ZIF nanozymes

**FIGURE 1:**
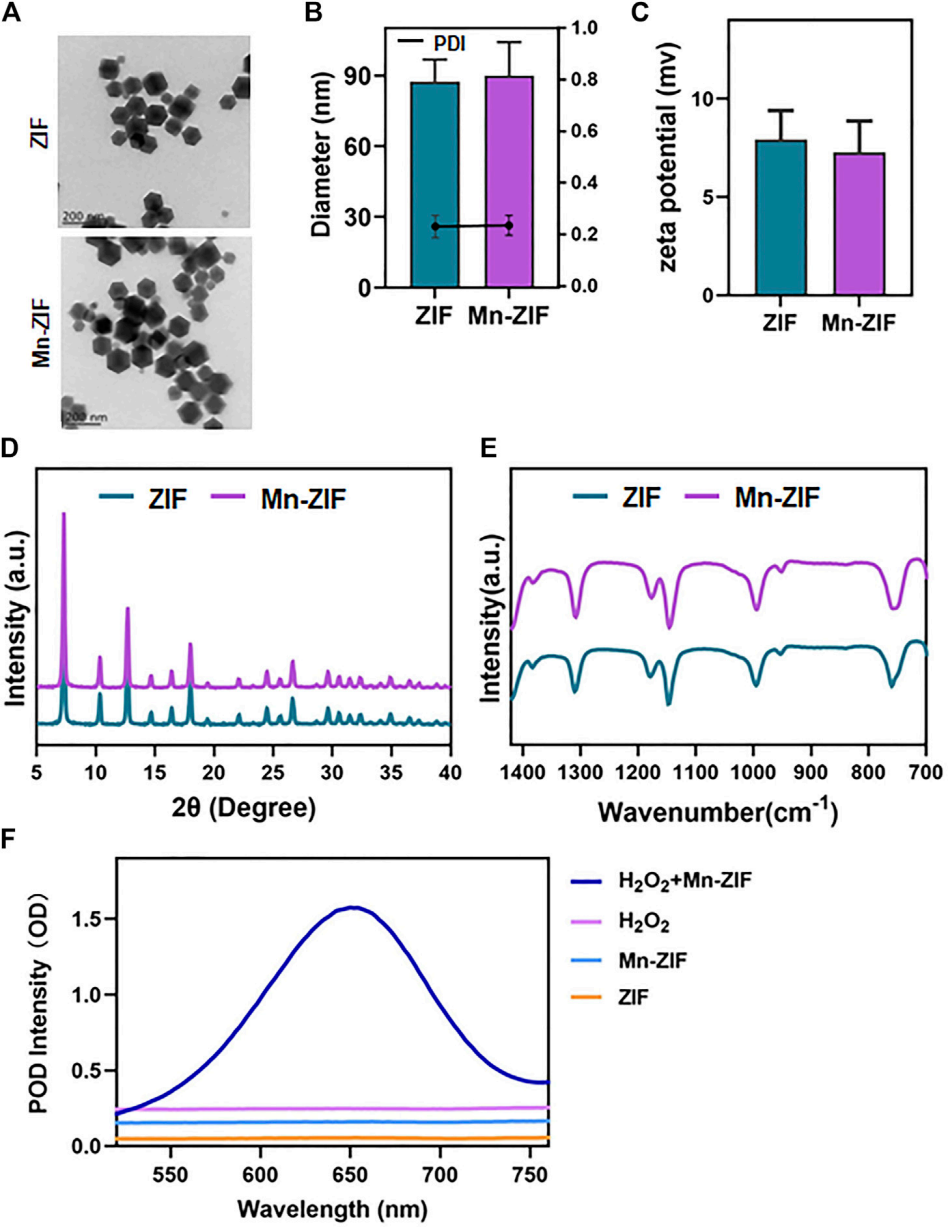
Characterization of nanozymes **(A)** SEM images of ZIF and Mn-ZIF. particle size **(B)** and potential **(C)** of different groups of nanoparticles. XRD **(D)** and FT-IR **(E)** results of different groups of nanoparticles. **(F)** POD activity of different groups of nanoparticles. Data are expressed as mean ± SD.

Based on previous experience, we believe that the synthesis of modified nano-enzymes by the one-pot method is a feasible option. The specific synthesis steps were described previously. We successfully synthesized ZIF nanozymes as well as modified Mn-ZIF nanozymes. First, we observed the morphology of the nanozymes by projection electron microscopy (TEM) (Figure 1A). We found that they all possessed the classical ZIF structure, showing an octahedral structure. After that, we measured the particle size of the nanoparticles with a Malvern instrument (Figure 1B). The diameter of the nanoparticles in the ZIF group was 87.3 ± 9.4 nm, whereas the diameter of the Mn-ZIF group was 89.9 ± 14.4 nm. This result suggests that both of them can increase the accumulation in the tumor region through the EPR effect. The results of the zeta potential showed that the potential of the ZIF group was 7.9 ± 1.5 mV, whereas the diameter of the Mn-ZIF group was 7.3 ± 1.6 mV (Figure 1C). They both carried trace amounts of positive charge. This could facilitate the interaction of the nanozymes with negatively charged cell membranes and organelles. This may enhance their distribution and permeability in the organism.

After that, we examined the crystal structures of both by XRD (Figure 1D). The results showed that the Mn-doped material retained the characteristic peaks compared to the classical ZIF structure. Meanwhile, we chose to further characterize the crystal morphology of both by Fourier transform infrared spectroscopy (Figure 1E). N-H oscillations can be observed, expanding at 1,313 cm^−1^. Methyl bends at 1,384 cm^−1^. In addition, C-N stretching and N-H oscillation at 1,181 cm^−1^. C=N out-of-plane bending and N-H bending at 762 cm^−1^ also appeared on different nanoparticles. This proves the feasibility of preparing Mn-ZIF by one-pot method. Moreover, the doping of Mn did not cause any damage to the crystalline morphology. Finally, we verified the POD enzyme activity of the materials (Figure 1F). We observed that in the presence of H_2_O_2_ as a substrate, ZIF still did not possess catalytic ability. In contrast, active POD enzyme activity was present in the modified Mn-ZIF nanozymes. H_2_O_2_ was catalyzed to ·OH by Fenton reaction. This again proved the successful preparation of Mn-ZIF nanozymes.

### Cytotoxicity and cellular uptake capacity of Mn-ZIF nanozymes

**FIGURE 2:**
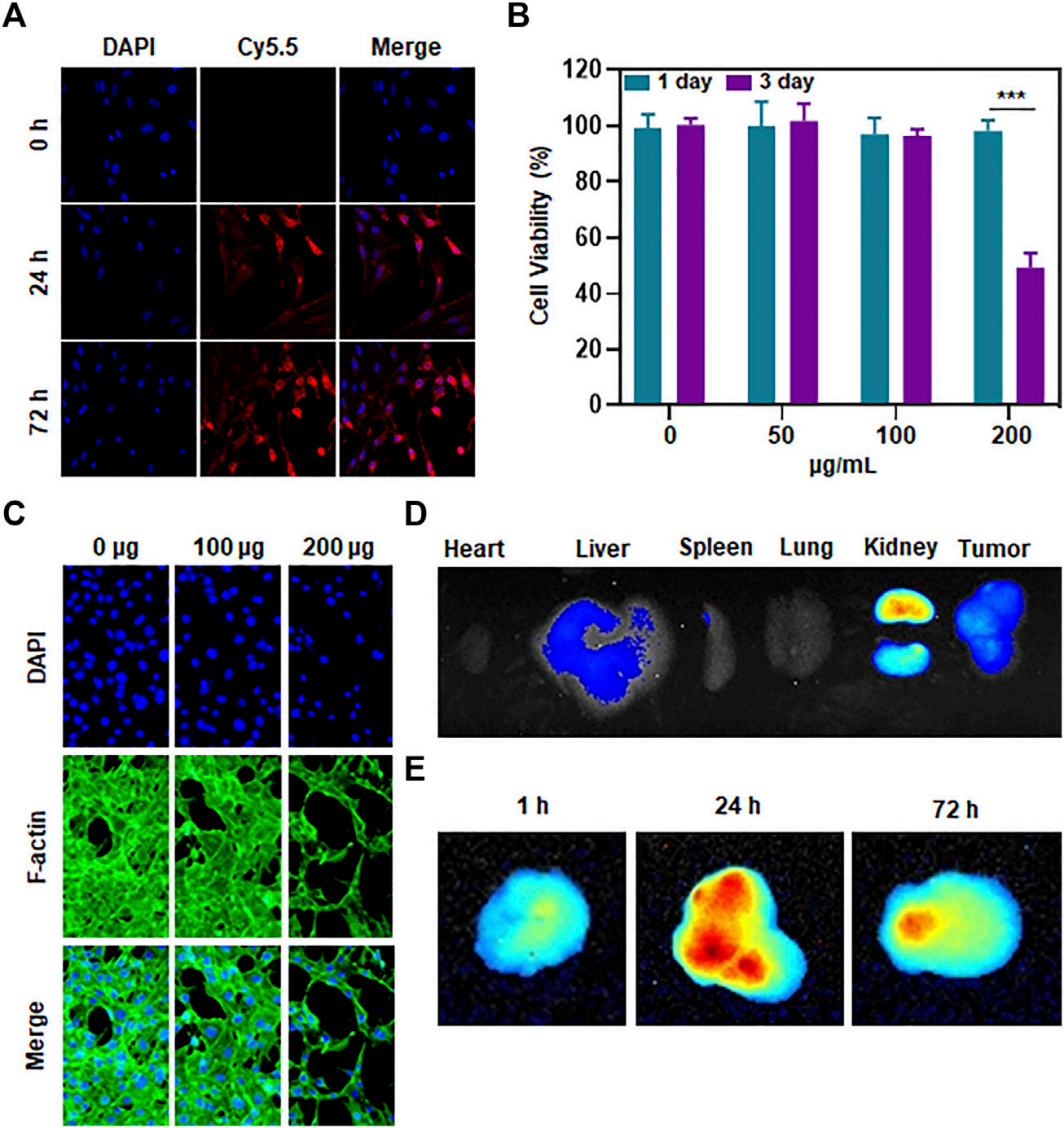
Penetration, toxicity and biodistribution of Mn-ZIF nanozymes on tumor cells **(A)** Uptake of material by tumor cells at different incubation durations. **(B)** Killing effect of different concentrations of materials on tumor cells. **(C)** Effect of different concentrations of materials on tumor cell growth inhibition. **(D)** Distribution of materials in different organs. **(E)** Content of materials in different time-length tumor regions. Data are expressed as mean ± SD; ***P < 0.005.

As mentioned earlier, we have paid great attention to the ability of Mn-ZIF nanozymes to penetrate tumor cell membranes. Whether it can successfully enter into tumor cells is a critical step in nanomaterials tumor therapy. We validated it at the cellular level. We labeled the Mn-ZIF nanozymes by using CY5.5. Afterwards, it was co-cultured with 143B cells to observe their uptake of Mn-ZIF nano-enzymes (Figure 2A). The results showed that before co-culture, the cells did not possess fluorescence intensity. And after 24 h of co-culture, there was a bright red fluorescence in the cytoplasm of 143B cells. This proved the excellent endocytosis of Mn-ZIF nano-enzymes. And the intensity of red fluorescence was further enhanced after co-culture up to 72 h. This proved the sustainability of the endocytosis. After 72 h of co-culture, we took CCK-8 to test the survival of cells after treatment with different concentrations (Figure 2B). When the concentration of Mn-ZIF reached to be 200 μg/mL, the proliferation of 143B cells was significantly inhibited. And at 100 μg/mL, there was no significant change in cell survival. We verified this result again by cell proliferation assay (Figure 2C). We observed a trend consistent with the previous one. The proliferation ability of tumor cells decreased minutely when the concentration of Mn-ZIF reached to 200 μg/mL. Finally, we demonstrated the distribution and metabolism of Mn-ZIF nanozymes in all major organs and tumor regions in mice by *in vivo* imaging. It is well known that the *in vivo* distribution of nanomedicines is critical for their therapeutic efficacy and speculation of possible side effects. We took the tail vein injection of Mn-ZIF that had undergone FITC labeling. And removed all major organs 24 h after injection, and resected the subcutaneous tumors at 1 h, 24 h, and 72 h after injection, respectively (Figures 2D, E). The results showed that Mn-ZIF nanozymes were mainly distributed in the liver, kidney and tumor regions. This suggested its possible metabolic pathway. In stripping and fluorescence imaging of tumors with different injection durations, it was found that the fluorescence intensity of the tumor region in the 24 h group was significantly stronger than that in the 1 h group. This was attributed to the EPR effect induced by the size of the Mn-ZIF nano-enzyme. And after 72 h we observed that the tumor region still retained some fluorescence intensity, suggesting the long retention effect of the nanomedicine. These data are critical for designing *in vivo* therapeutic regimens.

### Therapeutic efficacy of Mn-ZIF nano-enzymes in a 143B tumor model

**FIGURE 3:**
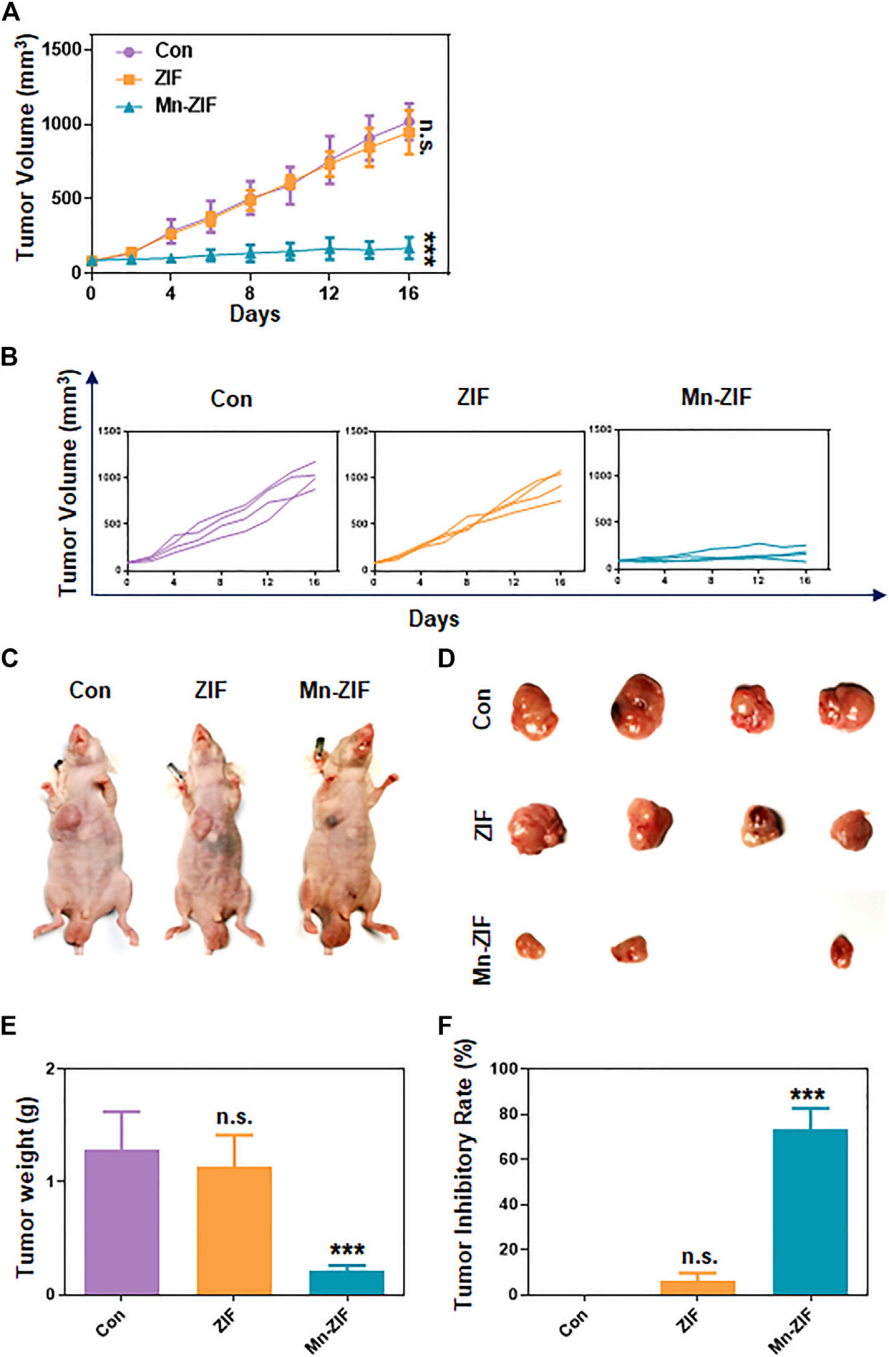
*In vivo* anti-tumor effects. Pooled tumor inhibition curves **(A)** and grouped tumor inhibition curves **(B)** for different materials. *In vivo* photographs **(C)** and *ex vivo* photographs **(D)** of tumors in each group of mice. Tumor weight **(E)** and tumor inhibition rate **(F)** of mice in each group. Data are expressed as mean ± SD; ***P < 0.005.

We took the subcutaneous 143B human osteosarcoma cell model in nude mice to observe the therapeutic effect of Mn-ZIF nanozymes on tumors. First, we measured the length and diameter of the subcutaneous tumors every 2 days to calculate the subcutaneous tumor volume, and plotted the summarized tumor growth curves (Figure 3A) and the tumor growth curves of each group of mice (Figure 3B). The results showed that the subcutaneous tumors in both the control and ZIF groups showed a faster growth trend. At the end of the treatment, the average tumor volume reached 1,018.6 mm^3^ and 946.8 mm^3^, respectively, while the average tumor volume in the Mn-ZIF group was only 171.3 mm^3^, and one of the tumors had nearly disappeared. This directly demonstrated the excellent anti-tumor effect of Mn-ZIF nanozymes. For the mice at the end of treatment, the researchers executed them and selected representative individuals for photographic display (Figure 3C). We could see the trend of tumor treatment consistent with the summarized tumor growth curves. In addition for all the tumor-bearing mice we isolated the tumor tissues in their bodies for photographing (Figure 3D). It can be clearly seen that the growth of tumor tissues in the Mn-ZIF nano-enzymes group is retarded and even the treatment has the effect of reducing the local tumors. These effects were not present in the ZIF alone group. Finally we weighed the weight of the tumors in each group and plotted the tumor inhibition rate (Figures 3E, F). These two sets of data allow us to draw conclusions consistent with the above.

### Mn-ZIF nanozymes can modulate the tumor immune microenvironment while killing tumors

**FIGURE 4:**
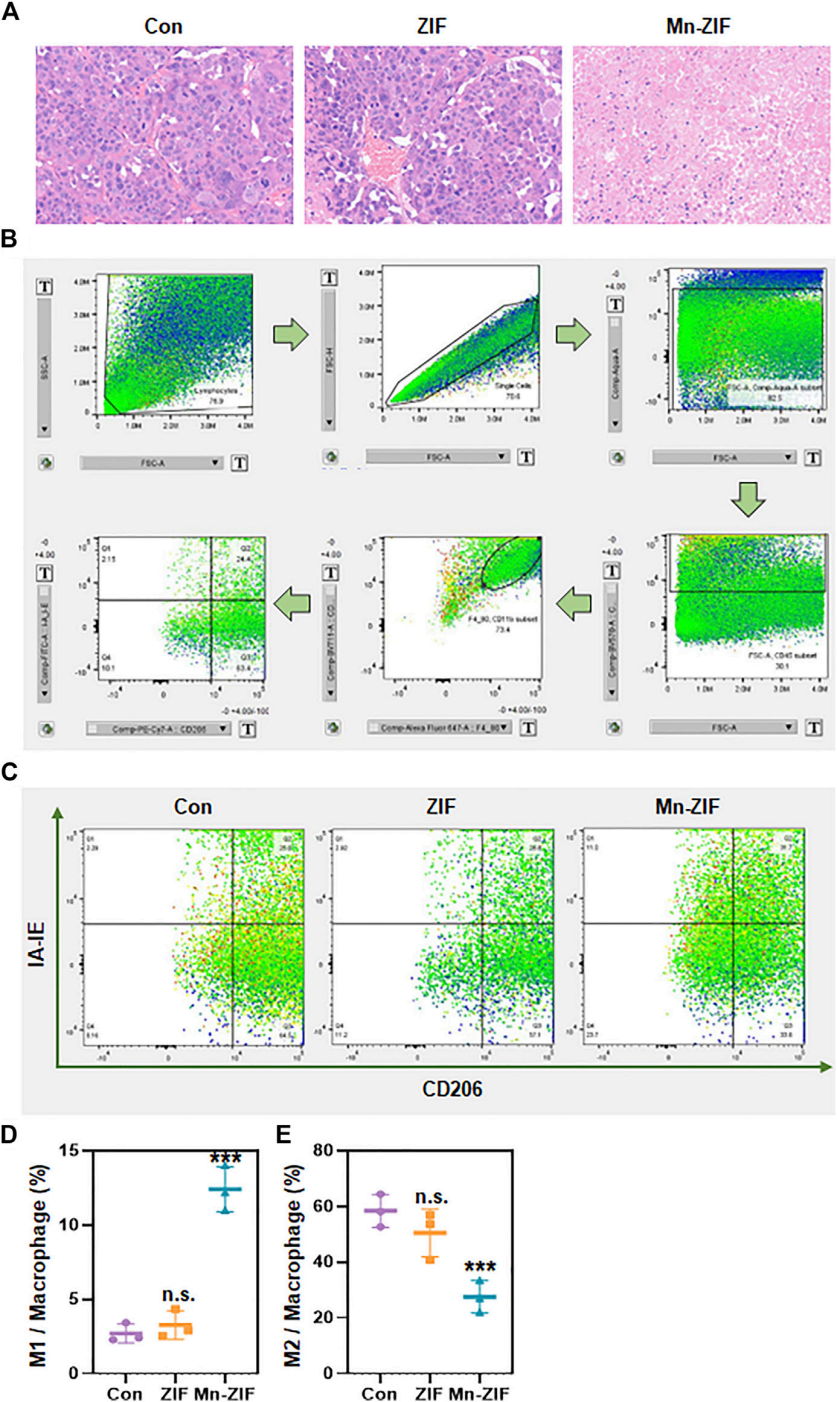
*In vivo* anti-tumor principles. **(A)** H&E staining of tumor regions in different groups. **(B)** Flow cytometry cell screening process. **(C)** Representative pictures of flow cytometry in different groups. **(D,E)** Statistics of flow cytometry results of different groups. Data are expressed as mean ± SD; ***P < 0.005.

In response to the above excellent efficacy of Mn-ZIF nano-enzymes for tumor inhibition *in vivo*, we further explored the principles involved. Firstly, we took the method of H&E staining of sections to further observe the differences in the pathological changes of the tumor areas between different groups (Figure 4A). We could observe that in the Con and ZIF groups, the tumor cells grew densely and had a healthy morphology. While in the Mn-ZIF group there were large areas of necrotic tissue in the tumor region. This suggests the occurrence of extensive tumor cell death. This result is consistent with the previous cellular experimental level verified that Mn-ZIF nanozymes can generate hydroxyl radicals to achieve direct tumor killing through Fenton reaction. And in previous work, some of the Mn-containing nanoparticles were prepared for local macrophage modulation. So in this study, we also took the means of flow cytometry to observe the macrophage changes in the tumor region. Where the step-by-step process of analyzing the results after flow cytometry is shown in the figure (Figure 4B). Typical flow cytometry results for each group were selected for presentation (Figure 4C). We took the same parameters to analyze the proportion of macrophages in the tumor area of three mice in each group (Figure 4D). From this, we were pleased to find that the proportion of M1-type macrophages in the tumor region reached more than 5-fold of the proportion in the Con group in the Mn-ZIF group. While no significant change was seen in the M1-type macrophage proportion in the ZIF group. Meanwhile, for the observation of the proportion of M2 type macrophages we found that the proportion in the Mn-ZIF group was much lower than that in the Con and ZIF groups. The results indicated that the polarization state of local macrophages was reversed after injection of Mn-ZIF nanozymes. Combined with the well-known effects of M1-type macrophages on tumor suppression and M2-type macrophages on tumor promotion, it is reasonable to believe that this effect on the repolarization of local macrophages in tumors can effectively assist in tumor therapy. Thus, we can achieve the purpose of combined oxidative stress and immunomodulation in the treatment of tumors.

### *In vivo* safety analysis of Mn-ZIF nano-enzymatic materials

**FIGURE 5:**
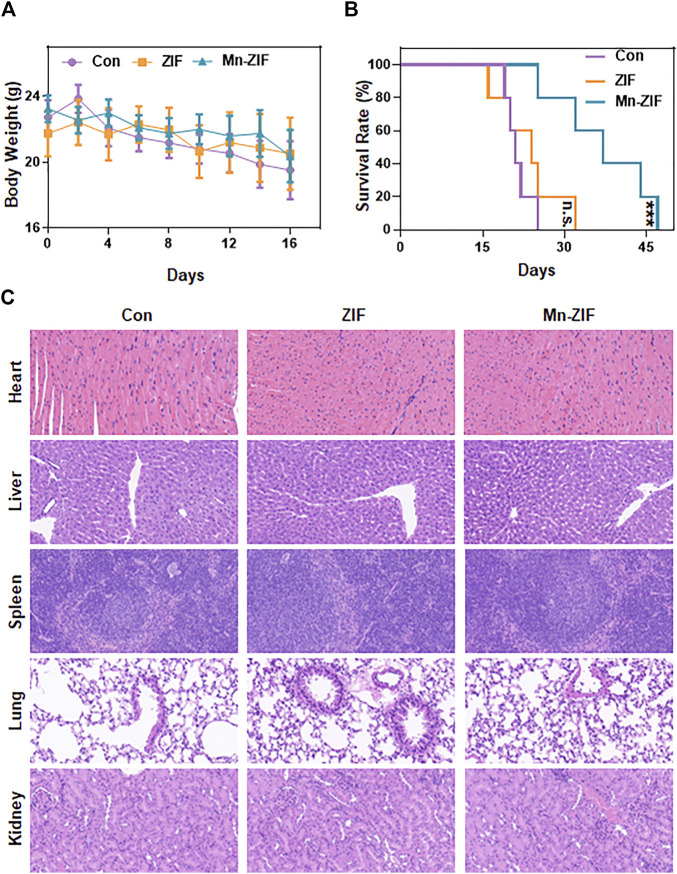
*In vivo* safety analysis. Body weight **(A)**, survival time **(B)**, and H&E sections of major organs **(C)** in different groups of mice. Data are expressed as mean ± SD; ***P < 0.005.

Finally, we observed the biosafety of the nanomaterial. First, the body weight data of each group of mice were measured and recorded throughout and the body weight curves were plotted (Figure 5A). The results showed that the body weights of the mice did not show any drastic changes regardless of whether the Mn-ZIF intervention was taken or not. This fully demonstrated the excellent biocompatibility of the material. We took the same method of drug administration as before and observed the survival cycle of the mice. The results were satisfactory. We found that the survival lifespan of mice intervened with Mn-ZIF was significantly longer than that of mice in the Con and ZIF groups (Figure 5B). This proves once again that Mn-ZIF nanozymes can effectively prolong the expected life span of patients while killing tumors. And this process is safe. In the previous we observed that Mn-ZIF is mainly metabolized by liver and kidney. Therefore, we took the method of H&E staining of tissue sections to observe the health of important organs in each group of mice (Figure 5C). The results showed that in the major organs of the three groups of mice, there was no pathological manifestation of organ damage. This again demonstrated the excellent biosafety of Mn-ZIF. This result provides favorable support for the clinical translational value of this material.

## Conclusion

In conclusion, Mn-doped ZIF nanozymes materials were successfully prepared. The Mn-ZIF nanozymes possess excellent POD enzyme activity. It can effectively oxidize H_2_O_2_ to hydroxyl radicals by catalyzing the occurrence of Fenton reaction. This effect can achieve tumor cell killing *in vitro*. In *in vivo* experiments, by passively targeting the tumor region, Mn-ZIF can effectively achieve the direct killing effect on tumor tissues. At the same time, the polarization state of tumor local macrophages was also reversed by the presence of Mn elements. By analyzing the results we found that both the production of POD activity and the changes in macrophage polarization are inextricably linked to the doping of Mn elements. This implies that Mn as an active element should be taken more into account in the future design of nanoenzymes. This two-for-one design provides a new idea and direction for the development of drugs for the comprehensive treatment of tumors.

## Data Availability

The original contributions presented in the study are included in the article/Supplementary Material, further inquiries can be directed to the corresponding authors.
