# Supplementary material for: Mn-ZIF nanozymes kill tumors by generating hydroxyl radical as well as reversing the tumor microenvironment
Source: Front Pharmacol. 2024 Aug 13;15:1441818. doi: 10.3389/fphar.2024.1441818 (PMC11347784; doi:10.3389/fphar.2024.1441818)
Supplement: Supplementary file 1 [file DataSheet1.docx]

Supplementary Material

Mn-ZIF nanozymes kill tumors by generating superoxide anion as well as reversing the tumor microenvironment

Jiyu Han^1,2†^, Hairong Ma^3†^, Songtao Ai^3*†^, Daqian Wan^1,2*†^

^1^ Department of Orthopedics, Tongji Hospital, School of Medicine, Tongji University, Shanghai 200065, China

^2^ Key Laboratory of Spine and Spinal Cord Injury Repair and Regeneration, Ministry of Education, Shanghai 200065, China

^3^Department of Radiology, Shanghai Ninth People’s Hospital, Shanghai Jiao Tong University School of Medicine, Shanghai 200011, China

†These authors have contributed equally to this work and share first authorship

# *†These authors have contributed equally to this work and share corresponding author

# Materials and methods

# Material, Cells, and Animals

Dulbecco's Modified Eagle Medium (DMEM) was purchased from Thermo Scientific. Fetal bovine serum was obtained from Sijiqing Company. 143B osteosarcoma cells were obtained from the Cell Bank of Shanghai Institute of Life Sciences, Chinese Academy of Sciences, and grown in high-glucose DMEM supplemented with 10% FBS, 100 μg/mL streptomycin, and 100 U/mL penicillin. The 143B cells were cultured at 37°C, 5% CO2, and a humid atmosphere. Male Balb/C nude mice (6 weeks old) were purchased from Beijing Vital River Laboratory Animal Technology Co., Ltd.Cell uptake

# Cell Uptake

Mn-ZIF was mixed with Cy5.5 to obtain Mn-ZIF-Cy5.5. 143B cells were co-cultured with Mn-ZIF-Cy5.5 for 0, 24, and 72 hours. After washing the cells with PBS and fixing them with 4% paraformaldehyde, cell uptake was observed using an inverted fluorescence microscope.

# In vitro Cell Toxicity Assay

Cell toxicity of Mn-ZIF was evaluated using a CCK-8 assay. After 143B cells adhered to the wall, they were incubated with DMEM containing different concentrations of Mn-ZIF for 1 and 3 days. Then, CCK-8 solution was added and incubated for 2 hours. The absorbance was measured at 450 nm using an enzyme-linked immunosorbent assay reader.

# 143B model construction in mice

Male Balb/C nude mice (6 weeks old) were anesthetized by intraperitoneal injection of 4 mg/mL pentobarbital sodium (60 μL). 143B cell suspension (100 μL containing 2 million cells) was injections into the chest under the skin. The needle was withdrawn, and pressure was applied to the injection site.

# Survival analysis

A total of 15 mice with a tumor volume of 80 mm3 were randomly divided into Control, ZIF, and Mn-ZIF groups, with 5 mice in each group. The treatment regimen was the same as before. The survival status of the mice was observed for survival analysis.

# Distribution and Metabolism of Materials In vivo

FITC-loaded Mn-ZIF was intravenously injected at a dose of 200 mg/kg in 200 μL. Mice were euthanized at 1, 24, and 72 hours post-injection, and the heart, liver, spleen, lungs, and kidneys were collected for fluorescence imaging. The fluorescence images of each organ in each mouse were captured using a small animal live imaging system (INDEC BioSystems, USA)

# H&E Staining and Imaging

The heart, liver, spleen, lungs, and kidneys collected from each group fixed with paraformaldehyde were embedded and sectioned. The tumor and organ sections were stained with H&E, and the tumor tissue as well as the heart, liver, spleen, lungs, and kidneys were observed under a Nikon Eclipse Ti microscope at 20x magnification for histological morphology.

# Statistical Analysis

Statistical analysis was performed using SPSS 18.0 (IBM, USA) software. Data were analyzed using Student's t-test. A p-value < 0.05 was considered statistically significant (95% confidence interval). Data are presented as mean ± standard deviation (SD).
